# MLpronto: A tool for democratizing machine learning

**DOI:** 10.1371/journal.pone.0294924

**Published:** 2023-11-30

**Authors:** Jacob Tjaden, Brian Tjaden

**Affiliations:** 1 Computer Science Department, Colby College, Waterville, ME, United States of America; 2 Department of Computer Science, Wellesley College, Wellesley, MA, United States of America; Brigham Young University, UNITED STATES

## Abstract

The democratization of machine learning is a popular and growing movement. In a world with a wealth of publicly available data, it is important that algorithms for analysis of data are accessible and usable by everyone. We present MLpronto, a system for machine learning analysis that is designed to be easy to use so as to facilitate engagement with machine learning algorithms. With its web interface, MLpronto requires no computer programming or machine learning background, and it normally returns results in a matter of seconds. As input, MLpronto takes a file of data to be analyzed. MLpronto then executes some of the more commonly used supervised machine learning algorithms on the data and reports the results of the analyses. As part of its execution, MLpronto generates computer programming code corresponding to its machine learning analysis, which it also supplies as output. Thus, MLpronto can be used as a no-code solution for citizen data scientists with no machine learning or programming background, as an educational tool for those learning about machine learning, and as a first step for those who prefer to engage with programming code in order to facilitate rapid development of machine learning projects. MLpronto is freely available for use at https://mlpronto.org/.

## Introduction

### Background and related work

With the broad availability of data to scientists and citizens alike, one of the challenges is extracting new insights from this wealth of data. Machine learning methods offer powerful tools for analyses of these rich datasets, and enable data-intensive, evidence-based predictions and decision making [1]. While many machine learning tools require expertise or access to significant computational resources to take advantage of them, many other tools have few if any barriers to their effective use, thanks, in part, to a community effort to democratize machine learning. For example, systems that suggest programming code, such as GitHub’s Copilot, OpenAI’s ChatGPT, and Replit’s Ghostwriter, have been growing in popularity [2]. While not restricted to the domain of machine learning, these code generation systems can be used as a resource, with both positive and negative consequences [3], for those beginning machine learning endeavors. More specific to machine learning, platforms such as OpenML [4] and Kaggle [5] make sharing of machine learning datasets, algorithms, and experiments simple and accessible. Similarly, automated machine learning (AutoML) systems have the ability to generate end-to-end pipelines for machine learning analyses [6]. AutoML tools normally integrate in a unified pipeline a variety of stages of analysis, such as data cleaning, feature engineering, model generation and hyperparameter tuning, ensemble assembly, and evaluation. Many AutoML systems provide low-code or no-code approaches that obviate the need for machine learning expertise from the user. There are AutoML options from established companies, such as Microsoft’s Azure ML, Apple’s CreateML, Google’s AutoML and Teachable Machine, and Amazon’s SageMaker, as well as a plethora of open-source software options available to the machine learning community [7].

### MLpronto’s contribution toward democratizing machine learning

Continuing this community trend of increasing accessibility of machine learning, MLpronto is a tool for engaging with popular machine learning algorithms. It is designed to be especially user-friendly so as to be accessible to as many people as possible. No computer programming or machine learning expertise is necessary to use MLpronto. For a user with data to analyze, the time between the start of project development and obtaining initial analysis results with MLpronto is a matter of moments. Thus, in the spirit of democratizing machine learning, MLpronto minimizes barriers to its usage and provides a vehicle for rapid initial interaction with machine learning. An important benefit of democratizing machine learning is that it helps to address concerns related to bias and fairness [8]. Enabling greater access to machine learning tools increases the diversity of people engaged in machine learning projects and workflows, reflecting a broader range of perspectives and experiences, and contributes to the development of more inclusive and equitable machine learning systems [9]. MLpronto is not an AutoML system, complete with feature engineering, model selection, hyperparameter tuning, and ensemble construction. Instead, in support of democratizing machine learning, MLpronto prioritizes usability and interpretability, and our results suggest that this increased accessibility does not necessarily come at a cost to performance, as MLpronto’s results are comparable to those of other state-of-the-art systems. MLpronto is freely available with no login requirements at https://mlpronto.org, with the source code available under the MIT License on GitHub at https://github.com/btjaden/MLpronto.

## Methods

**Fig 1:**
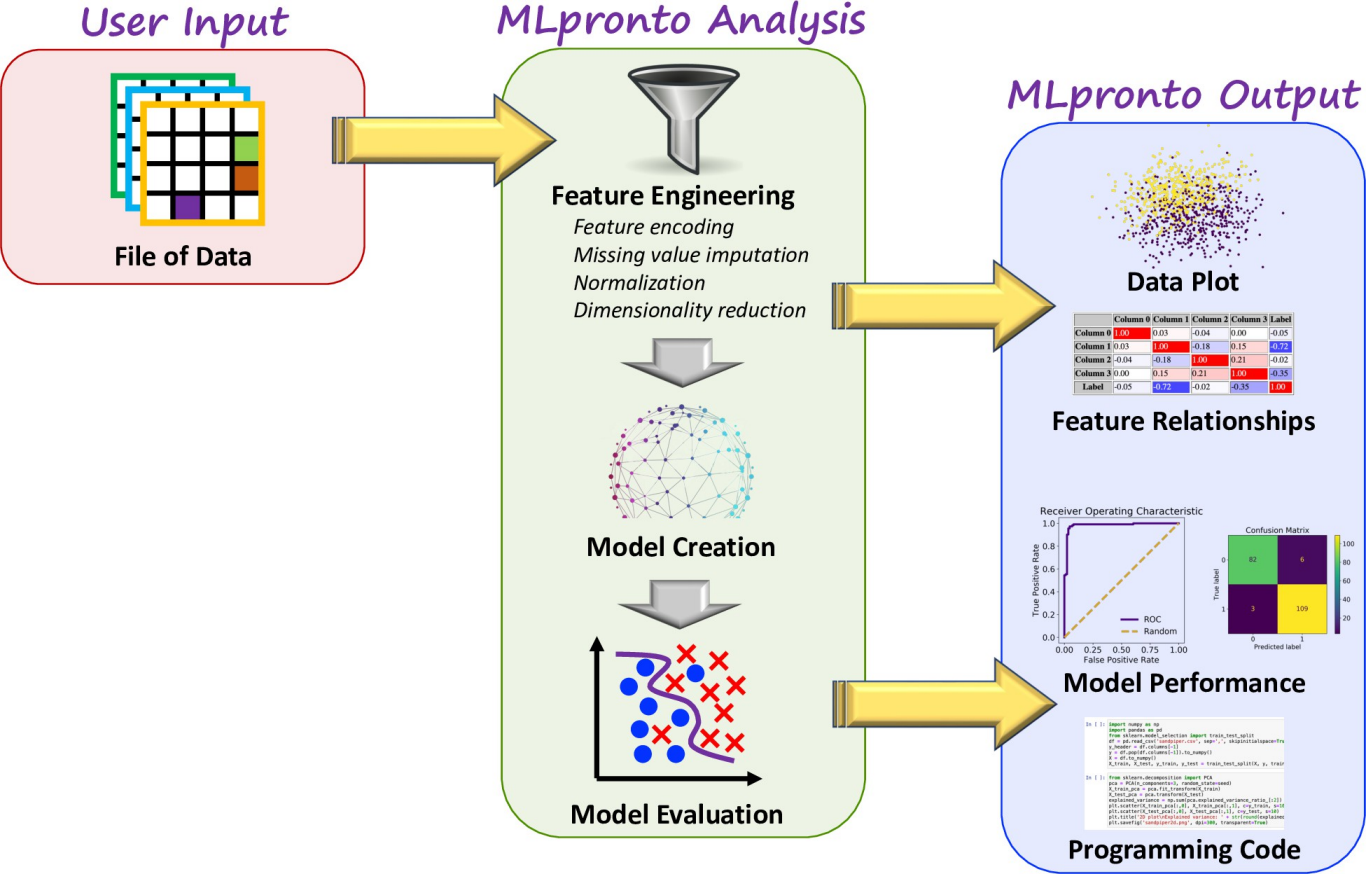
MLpronto workflow. The stages of MLpronto are depicted. The user supplies a file of structured data. MLpronto performs a variety of feature engineering steps before training a machine learning model and evaluating the model’s performance. Throughout the stages of its analysis, MLpronto outputs numerous quantitative and graphical representations of results.

MLpronto is implemented in Python and uses the scikit-learn library [10] to execute supervised classification and regression machine learning algorithms. The various stages of MLpronto and its workflow are illustrated in Fig 1. As input, MLpronto requires a file of structured data in any of several common formats, including text or spreadsheet. Alternatively, MLpronto provides example data files that may be used to immediately explore the functionality of MLpronto without the user having to provide any data. There are several parameters that may be specified, such as how missing data are handled, whether feature scaling should be used, and which specific learning algorithm to execute. Presently, MLpronto offers sixteen options for classification and regression algorithms.

As output, MLpronto provides results from a variety of analyses. First, it projects the data into 2 and 3 dimensional space via principal component analysis and plots the data, indicating the percentage of variance explained by the plotted principal components. Then it calculates several relationships among the features. The correlations between every pair of columns in the data are illustrated in a table. Similarly, the mutual information, f-statistic, and corresponding p-value are reported for each column with respect to the dependent variable. The f-statistic and p-value are based on ANOVA for classification analyses and on univariate linear regression for regression analyses. Various metrics are reported separately for training data and testing data. For classification analyses, these metrics include accuracy, F1 score, precision, recall, and area under the receiver operating characteristic (ROC) curve. For regression analyses, these metrics include R^2^ score, adjusted R^2^ score, out of sample R^2^ score (for testing data), mean squared error, and mean absolute error. In the case of classification analyses, additional results reported include the confusion matrix, classification report, a ROC plot and a precision-recall curve plot. In the case of regression analyses, additional results reported include a plot of predicted vs. actual values, a residuals vs. fits plot, and a histogram of residuals to provide some indication as to whether the error terms appear to be normally distributed.

As part of performing the abovementioned analyses, MLpronto generates programming code, specific to the input dataset and parameter options specified by the user. The generated code is reported as a Python file and as a Jupyter notebook. The parameter values are reported as a JSON file. The code files can be executed immediately by the user on their local system, assuming access to the appropriate Python libraries, so that the results reported by MLpronto can be reproduced and the analyses can be customized and built upon as part of evolving machine learning projects.

### Executing tools on benchmark datasets

We evaluated the performance of MLpronto and five other machine learning tools on the Penn Machine Learning Benchmarks (PMLB) [11, 12]. The PMLB datasets consist of 165 binary and multi-class classification problems and 121 regression problems corresponding to a wide range of applications. The five tools to which we compare MLpronto are Weka [13], PyTorch [14], Auto-Weka [15], Auto-PyTorch [16], and Auto-Sklearn [17, 18]. The first two tools, Weka and PyTorch, are single-model tools in which the user selects a single specific machine learning model. The latter three tools, AutoWeka, Auto-PyTorch, and Auto-Sklearn, are AutoML tools that build ensembles of machine learning models.

MLpronto and the five other tools were evaluated on the 286 benchmark datasets. For MLpronto, default parameter settings were used and, to avoid any performance optimization, the same algorithm, gradient boosting, was used on all datasets. For Weka, default parameter settings were used with the J48 algorithm. For PyTorch, default parameter settings were used with a neural network containing one hidden layer consisting of one hundred nodes, optimized with the Adam algorithm over 500 epochs with a loss function of binary cross entropy loss (for binary classification datasets), cross entropy loss (for multi-class classification datasets), or mean squared error loss (for regression datasets). For the three AutoML algorithms, default parameter settings were used except that the available memory was increased from 1–3 gigabytes (default) to 12 gigabytes. Whereas single-model tools such as MLpronto, Weka, and PyTorch run until they complete execution, AutoML tools run for a user-specified length of time, constantly exploring the search space and improving performance. Thus, the three AutoML tools were executed on each dataset for different lengths of time, ranging from the minimum time allowed by the tool up to 600 seconds.

## Results

In the spirit of democratizing machine learning, MLpronto can be run from a user-friendly web interface with no coding development necessary and rapid execution times. In order to evaluate to what extent MLpronto’s ease-of-use comes at the cost of accuracy in results, we compared MLpronto’s performance with that of five other advanced machine learning tools: Weka, PyTorch, Auto-Weka, Auto-PyTorch, and Auto-Sklearn (see Methods above). The first two, Weka and PyTorch, are single-model tools like MLpronto where the user selects a particular machine learning model to employ. PyTorch has a focus on deep learning models, and we used PyTorch to develop a neural network with a 100-node hidden layer for each dataset upon which it was run. PyTorch uses Python and Weka uses Java as its programming language. The latter three tools, AutoWeka, Auto-PyTorch, and Auto-Sklearn, are popular AutoML systems based on Weka, PyTorch, and scikit-learn, respectively. These AutoML systems use meta-learning and Bayesian optimization to determine the optimal learning algorithms and their associated hyperparameter optimizations in combined search spaces [19]. Thus, the three AutoML systems have different objectives than MLpronto, which is a single-model system rather than an AutoML system. For a given dataset, the aim of the AutoML systems is to optimize prediction accuracy on the dataset by determining the best possible ensemble of machine learning algorithms with their optimal hyperparameters. In contrast, MLpronto’s aim is to perform rapid and interpretable analyses, so that MLpronto is optimizing user-friendliness and accessibility. For example, MLpronto is a no-code tool, whereas AutoML systems normally require a background in machine learning programming. Despite the different emphases of MLpronto and AutoML systems, a comparison helps illuminate MLpronto’s strengths and limitations. For objective comparison, we evaluated MLpronto and the five other machine learning tools on PMLB, a large collection of curated benchmark datasets for assessing machine learning algorithms (see Methods above). The benchmark datasets contain 165 classification problems and 121 regression problems.

### MLpronto performance compared to single-model tools

**Fig 2:**
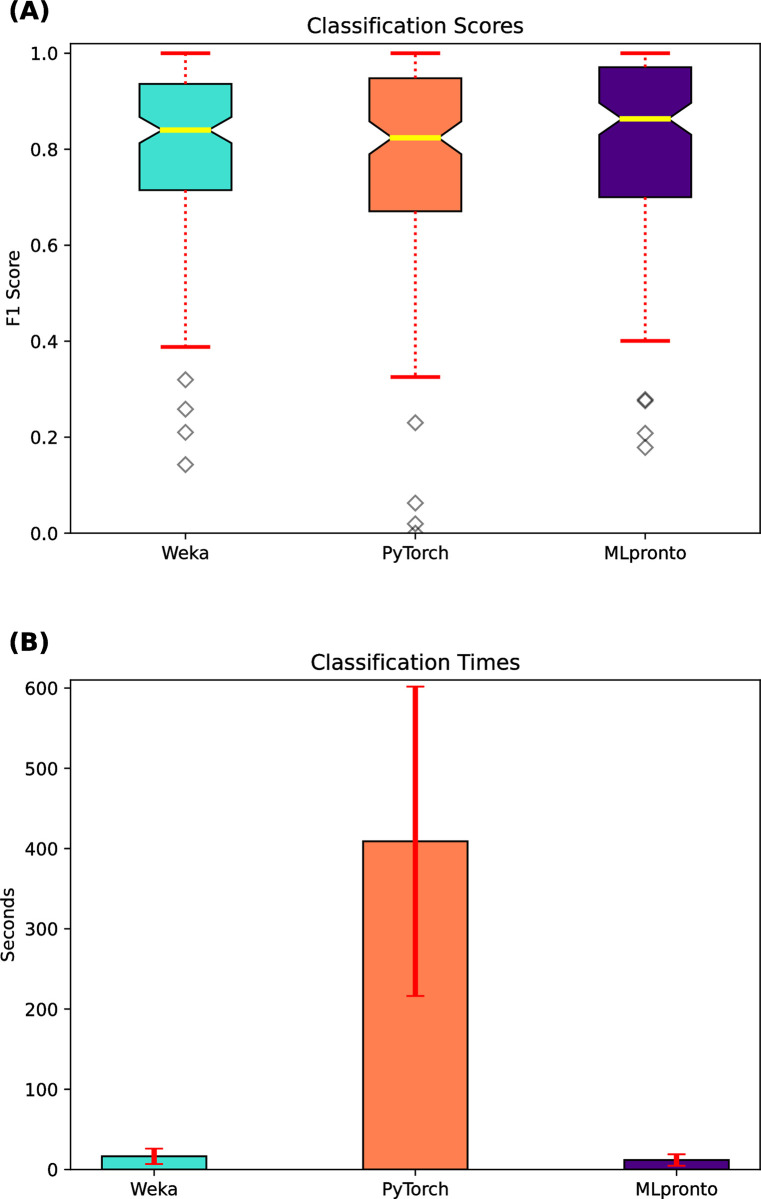
Classification performance of single-model tools. On 165 classification datasets, results are shown when executing three single-model tools, Weka, PyTorch, and MLpronto. **(A)** The box and whisker plot illustrates the performance of the tools as measured by F1 score. The horizontal line (yellow) indicates the median F1 score across the 165 classification datasets. The top and bottom of the box indicate the 25th percentile and the 75th percentile, respectively. The notches in a box represent the confidence interval around the median. The whiskers (red) extend from the box by 1.5 times the inter-quartile range. Outlier (flier) points are indicated as diamonds (gray). **(B)** The bar plot illustrates the mean runtime of training for each tool on the 165 classification datasets. Error bars (red) correspond to standard error.

For the 165 classification datasets, Fig 2A indicates the performance, as measured by F1 score, of Weka and PyTorch and MLpronto. Across the 165 datasets, the median F1 scores of Weka and PyTorch are 0.84 and 0.82, respectively, whereas the median F1 score of MLpronto is 0.86. There is no statistically significant (*p*-value < 0.01) difference between the mean values of the three distributions. Fig 2B shows the mean runtime of Weka and PyTorch and MLpronto across the 165 classification datasets. These results suggest that the three tools have comparable performance on the classification problems, and Weka and MLpronto generally operate with faster runtimes.

**Fig 3:**
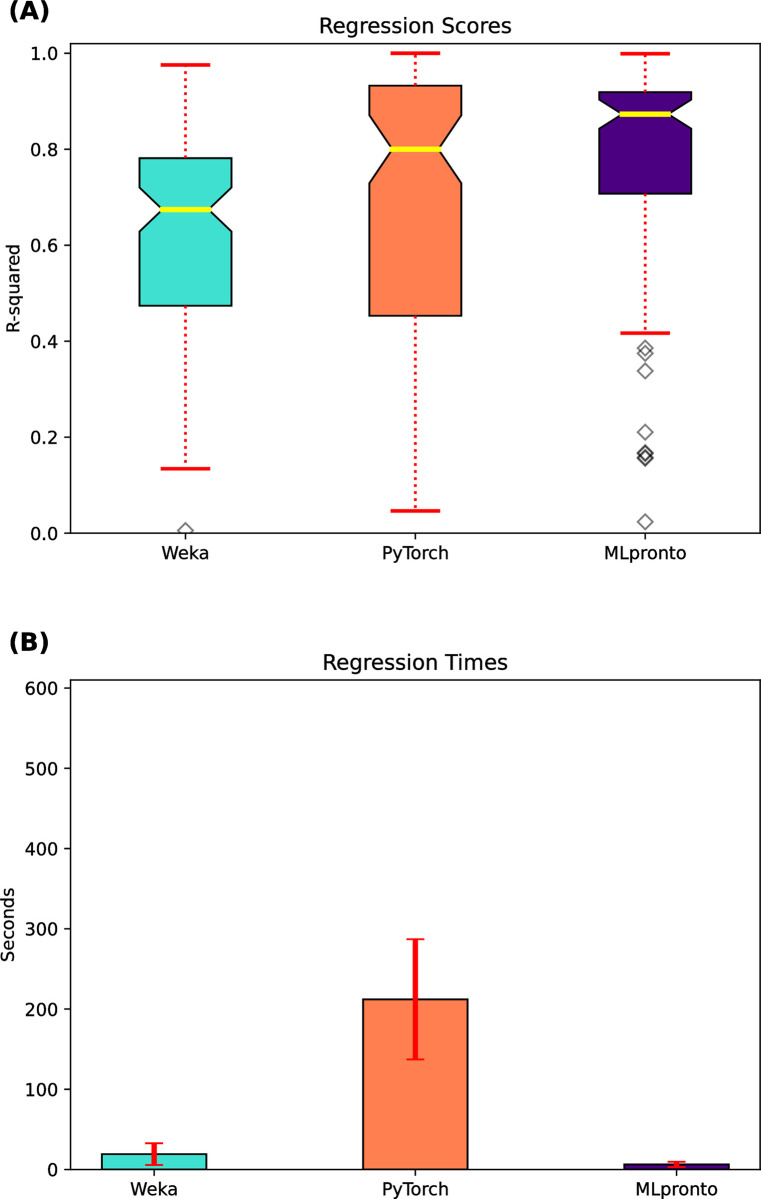
Regression performance of single-model tools. On 121 regression datasets, results are shown when executing three single-model tools, Weka, PyTorch, and MLpronto. **(A)** The box and whisker plot illustrates the performance of the tools as measured by R^2^ score. The horizontal line (yellow) indicates the median R^2^ score across the 121 regression datasets. The top and bottom of the box indicate the 25th percentile and the 75th percentile, respectively. The notches in a box represent the confidence interval around the median. The whiskers (red) extend from the box by 1.5 times the inter-quartile range. Outlier (flier) points are indicated as diamonds (gray). **(B)** The bar plot illustrates the mean runtime of training for each tool on the 121 regression datasets. Error bars (red) correspond to standard error.

For the 121 regression datasets, Fig 3A indicates the performance, as measured by R^2^ score, of Weka and PyTorch and MLpronto. Across the 121 datasets, the median R^2^ scores of Weka and PyTorch are 0.66 and 0.79, respectively, whereas the median R^2^ score of MLpronto is 0.87. There is a statistically significant difference between the mean value of MLpronto and that of Weka (*p*-value = 1.7e-5) and of PyTorch (*p*-value = 1.6e-3). Fig 3B shows the mean runtime of Weka and PyTorch and MLpronto across the 121 regression datasets. These results suggest that MLpronto has the best performance on the regression problems, and Weka and MLpronto generally operate with faster runtimes.

### MLpronto performance compared to AutoML tools

**Fig 4:**
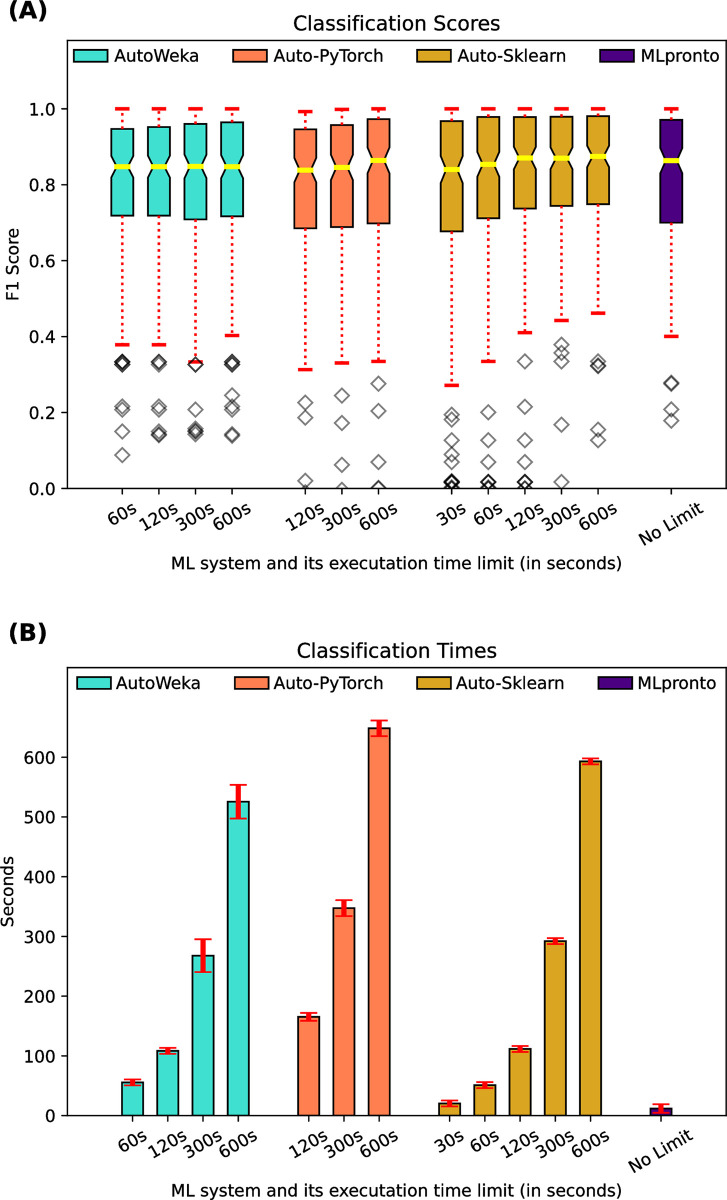
Classification performance of AutoML tools and MLpronto. On 165 classification datasets, results are shown when executing three AutoML tools, AutoWeka, Auto-PyTorch, Auto-Sklearn, for different lengths of time and when executing MLpronto. **(A)** The box and whisker plot illustrates the performance of the tools as measured by F1 score. The horizontal line (yellow) indicates the median F1 score across the 165 classification datasets. The top and bottom of the box indicate the 25th percentile and the 75th percentile, respectively. The notches in a box represent the confidence interval around the median. The whiskers (red) extend from the box by 1.5 times the inter-quartile range. Outlier (flier) points are indicated as diamonds (gray). **(B)** The bar plot illustrates the mean runtime of training for each tool on the 165 classification datasets. Error bars (red) correspond to standard error.

For the 165 classification datasets, Fig 4A indicates the performance, as measured by F1 score, of three AutoML tools, AutoWeka, Auto-PyTorch, and Auto-Sklearn, as compared to that of MLpronto. The three AutoML tools were executed for different lengths of time, ranging from the tool’s minimum runtime up to 600 seconds. Across the 165 datasets, the median F1 score of AutoWeka ranges from 0.85 when executed for 60 seconds to 0.85 when executed for 600 seconds. The median F1 score of Auto-PyTorch ranges from 0.84 when executed for 120 seconds to 0.86 when executed for 600 seconds. The median F1 score of Auto-Sklearn ranges from 0.84 when executed for 30 seconds to 0.87 when executed for 600 seconds. For comparison, the median F1 score of MLpronto is 0.86. There is no statistically significant (*p*-value < 0.01) difference between the mean values of these distributions. Fig 4B shows the mean runtime of the tools across the 165 classification datasets. These results suggest that the tools have comparable performance on the classification problems, and MLpronto generally operates with faster runtimes.

**Fig 5:**
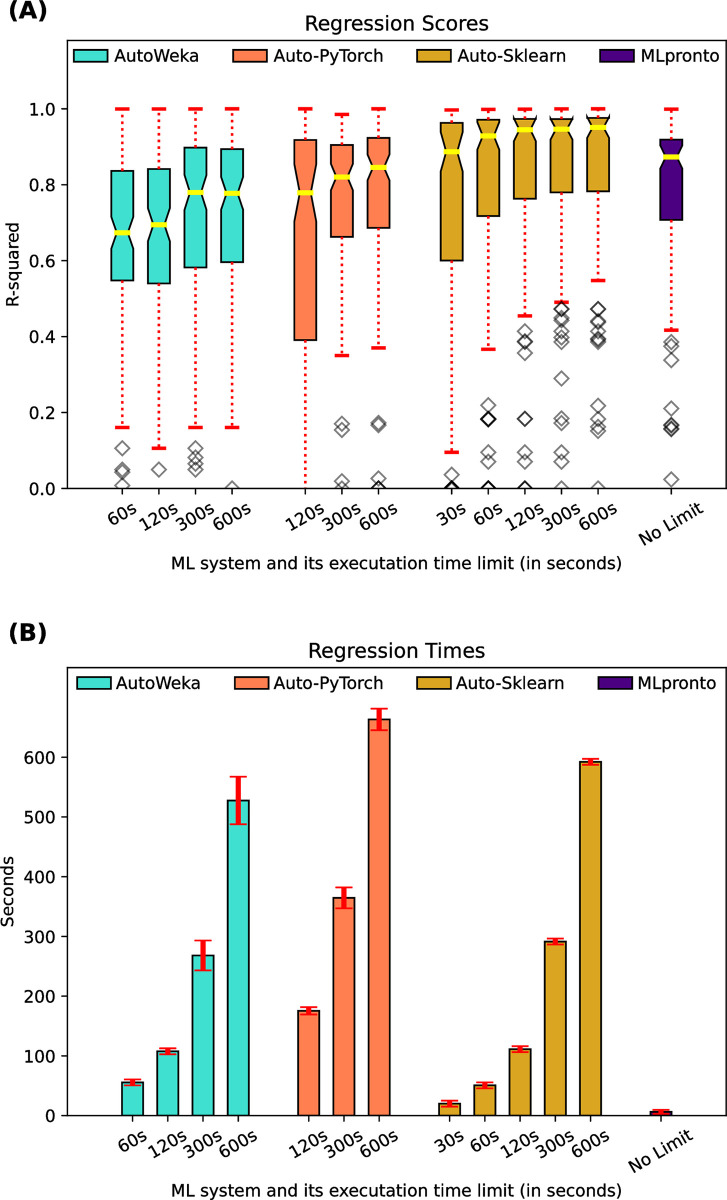
Regression performance of AutoML tools and MLpronto. On 121 regression datasets, results are shown when executing three AutoML tools, AutoWeka, Auto-PyTorch, Auto-Sklearn, for different lengths of time and when executing MLpronto. **(A)** The box and whisker plot illustrates the performance of the tools as measured by R^2^ score. The horizontal line (yellow) indicates the median R^2^ score across the 121 regression datasets. The top and bottom of the box indicate the 25th percentile and the 75th percentile, respectively. The notches in a box represent the confidence interval around the median. The whiskers (red) extend from the box by 1.5 times the inter-quartile range. Outlier (flier) points are indicated as diamonds (gray). **(B)** The bar plot illustrates the mean runtime of training for each tool on the 121 regression datasets. Error bars (red) correspond to standard error.

For the 121 regression datasets, Fig 5A indicates the performance, as measured by R^2^ score, of three AutoML tools, AutoWeka, Auto-PyTorch, and Auto-Sklearn, as compared to that of MLpronto. The three AutoML tools were executed for different lengths of time, ranging from the tool’s minimum runtime up to 600 seconds. Across the 121 datasets, the median R^2^ score of AutoWeka ranges from 0.67 when executed for 60 seconds to 0.78 when executed for 600 seconds. The median R^2^ score of Auto-PyTorch ranges from 0.78 when executed for 120 seconds to 0.85 when executed for 600 seconds. The median R^2^ score of Auto-Sklearn ranges from 0.89 when executed for 30 seconds to 0.95 when executed for 600 seconds. For comparison, the median R^2^ score of MLpronto is 0.87. There is a statistically significant difference between the mean value of MLpronto and that of Auto-Weka when run for 60 seconds (*p*-value = 6.5e-5) or 120 seconds (*p*-value = 5.2e-4), and of Auto-PyTorch when run for 120 seconds (*p*-value = 1.2e-4). There is no statistically significant (*p*-value < 0.01) difference between the mean values of MLpronto and of Auto-Weka or Auto-PyTorch when executed for longer periods of time or between the mean values of MLpronto and Auto-Sklearn for any runtime period. Fig 5B shows the mean runtime of the tools across the 121 regression datasets. These results suggest that the tools, at least when executed for long enough in the case of AutoWeka and Auto-PyTorch, have comparable performance on the regression problems. Auto-Sklearn performs the best, although the difference with MLpronto is not statistically significant (*p*-value of 0.052), even when Auto-Sklearn is executed for as much as 600 seconds. MLpronto operates with the fastest runtimes.

## Discussion

The goal of MLpronto is to enable everyone, including those with no machine learning or computer programming background whatsoever, to simply and quickly apply machine learning methods to their data. MLpronto executes some of the more common supervised machine learning algorithms on a set of data and provides the results via a user-friendly web interface, usually within a matter of seconds. For those users who prefer to engage with programming code, MLpronto also generates code that can be used for data analysis, customized, and built upon for expeditious development of machine learning projects. Thus, MLpronto can be used as a no-code tool for those new to machine learning, or as a starting point for rapid machine learning project iteration.

While MLpronto’s target audience is those at the beginning of their machine learning journey, in order to better understand its strengths and limitations, we compared MLpronto to mature tools that require expertise to utilize. We compared MLpronto to two popular single-model tools and three robust AutoML tools that, for a given dataset, optimize which machine learning algorithms to use, whether and how to preprocess the features, how to set all hyperparameters, and how to combine models into an ultimate ensemble prediction system. While MLpronto is designed with simplicity, efficiency, and user-friendliness at its core, the tools to which we compare MLpronto are designed with performance, e.g., maximizing predictive accuracy, at their core. All tools were executed on a suite of 286 benchmark datasets corresponding to classification and regression problems. Overall across the suite of benchmark datasets, MLpronto’s performance in terms of predictive accuracy was comparable to that of the expert tools. Only one tool, Auto-Sklearn, achieved higher accuracy than MLpronto, though only modestly so. However, MLpronto generally operates with substantially faster execution times and MLpronto requires no code development. For users with expertise whose goal is maximizing predictive accuracy, AutoML systems executed for longer runtimes may be the better option. For users who do not have the time, resources, or expertise with programming in a machine learning context, we offer MLpronto as a competitive option.

Indeed, it is somewhat surprising that MLpronto’s performance is comparable to that of mature AutoML systems. We hypothesized that the AutoML systems would show dramatically better performance than MLpronto since the AutoML systems, unlike MLpronto, are attempting to optimize a large search space of preprocessing methods, machine learning algorithms, and hyperparameter values. But this was not the case. We offer a couple of explanations for these results. First, the AutoML systems may best be suited for scenarios with much longer runtimes. We ran three AutoML tools for up to 10 minutes per dataset with performance improving with increasing runtime, though the performance generally begins leveling off as the runtime approaches 10 minutes. However, the authors of one AutoML system, Auto-Sklearn, have suggested a runtime of 24 hours for each dataset on which the system is executed [17]. This provides evidence, unsurprisingly, of the trade-off between performance (with respect to predictive accuracy) and efficiency (with respect to runtime), where AutoML systems better achieve the former and MLpronto the latter. Second, results are only as good as the data from which they are generated. The 286 PMLB datasets that we used for evaluation are composed of a large range of data from different domains, and to the extent that they are representative of datasets on which MLpronto is executed, the results we report here should be reasonably indicative of what users can expect. It is possible that results may differ for datasets that are very different from those found in PMLB.

### Limitations and future work

While MLpronto can be a useful tool in many contexts, it is important to understand its limitations. MLpronto focuses on mature supervised machine learning methods and, at least currently, not on unsupervised methods, hyperparameter tuning, architecture search, model selection, or data acquisition and wrangling. While all of these components are important parts of machine learning pipelines, there are other well-designed systems that support these components, such as from the AutoML community. MLpronto restricts its focus in order to maximize simplicity and usability for a broad audience. Relatedly, MLpronto is not designed for massive datasets, at least via the web server. The web server limits the size of input files to 100 megabytes. For users with larger datasets or with private datasets that ought not be uploaded, it is recommended that users download the source code, which has no restrictions on the size of input files. Further, at least presently, MLpronto restricts input data to be structured, e.g., in text or spreadsheet format, and not other forms, such as image files, audio files, or general unstructured text. Going forward, there are a number of directions that we plan to pursue for evolving MLpronto, including supporting more varied types of input as well as partial optimization of the hyperparameter search space, to the extent that MLpronto’s accessibility is not compromised. Additionally, we plan for MLpronto to output code not only in Python but also in R and other programming languages popular for machine learning in order to facilitate engagement in communities with disparate programming language preferences. Ultimately, our aim with MLpronto is contributing toward making machine learning usable by anyone and accessible to everyone.
